# Do Different Hurdle Heights Alter Important Spatiotemporal Variables in Hurdle Clearance?

**DOI:** 10.3389/fspor.2022.822592

**Published:** 2022-03-10

**Authors:** Yusuke Ozaki, Takeshi Ueda

**Affiliations:** Graduate School of Humanities and Social Sciences, Hiroshima University, Higashihiroshima, Japan

**Keywords:** hurdle event, sprint, athletics, coaching, track and field

## Abstract

The aim of this study was to determine whether important spatiotemporal variables in hurdle clearance change with different hurdle heights. Twelve male hurdlers (mean height, 1.75 ± 0.04 m) cleared hurdles set at different heights [10% higher (High) and 10% lower (Low) than the center of mass (CM)]; images were captured by six high-speed cameras, and each spatiotemporal variable was calculated. Thereafter, the difference in each spatiotemporal variable between High and Low and the relationship between the mean horizontal velocity from takeoff to landing [Hurdle clearance velocity (HC-v)] and each spatiotemporal variable were examined. Our findings indicated that values for flight time, flight time from hurdle to landing (2nd flight time), clearance time, release height on takeoff, peak height of the CM, and the difference in landing distance were greater in the High condition than in the Low condition. Moreover, a low rate of deceleration on takeoff and short 2nd flight time, clearance distance, and takeoff distance were more strongly related to HC-v in the condition High, whereas a low rate of deceleration on landing, short flight time from takeoff to hurdle (1st flight time), high release height on landing, and touchdown height on landing were more strongly related to HC-v in the Low condition. Therefore, coaches should consider these changes in spatiotemporal variables when changing hurdle heights based on age group or event. It should also be noted that, even when the hurdle heights are the same, the spatiotemporal variables that should be considered may differ depending on the height of the hurdler.

## Introduction

The hurdle events in track and field [100 m hurdles (mH), 110 mH, 400 mH, and 60 mH] are events in which runners compete for time by clearing hurdles of specified heights placed at equal intervals. Hurdle clearance is one of the most representative techniques of hurdle events, and the kinematic and spatiotemporal characteristics of hurdle clearance in high-performance hurdlers have been clarified (Mann and Herman, 1985; McDonald and Dapena, 1991a; McLean, 1994; Salo et al., 1997; McDonald, 2002; Čoh, 2003; Iskra and Coh, 2006, 2011; Čoh and Iskra, 2012; Coh et al., 2019; Čoh et al., 2020; González-Frutos et al., 2019; Hanley et al., 2021).

However, in hurdle events, the hurdle height and interval distance differ between men (110 mH, height: 1.067 m, interval: 9.14 m) and women (100 mH, height: 0.838 m, interval: 8.50 m). McDonald and Dapena (1991a) showed that men have a larger takeoff angle, larger rate of deceleration on takeoff, and a peak height of the center of mass (CM) that appears closer to the back of the hurdle than women. A study on world championship finalists also reported that the distance from the takeoff position to the hurdle (takeoff distance) was longer for men than for women, but when normalized for hurdle height, it was longer for women than for men (Hanley et al., 2021).

Regarding the relationship of spatiotemporal variables to performance, Čoh and Iskra (2012) reported the following characteristics of high-performance male hurdlers: short support time on takeoff, short braking phase and long propulsion phase on takeoff, long takeoff distance, short distance from the hurdle to the landing position (landing distance), short flight time, short braking phase on landing, and high CM height on landing. Salo et al. (1997) reported that takeoff distance did not differ according to performance in men; however, in women, the higher the performance, the longer the takeoff distance. Additionally, in a study on the 60 mH event, faster race time was associated with a longer takeoff distance and shorter landing distance in men; however, this was not the case in women (González-Frutos et al., 2019). Thus, the spatiotemporal variables that are important in hurdle clearance are not always consistent between men and women and may differ according to performance level.

Discrepancies in the results of previous studies that examined spatiotemporal variables in hurdle events (McDonald and Dapena, 1991a; Salo et al., 1997; Čoh and Iskra, 2012; González-Frutos et al., 2019; Hanley et al., 2021) may have been influenced by differences in body size, sprint ability, interval distance, and hurdle height. However, the main factors that cause differences in important spatiotemporal variables in hurdle clearance have yet to be elucidated. Therefore, to clarify these factors, it is necessary to examine the effect of hurdle heights on spatiotemporal variables in hurdle clearance while controlling for confounding variables.

In short-sprint hurdle events, the hurdle height gradually increases with each age group (U18: 0.914 m for men, 0.762 m for women; U20: 0.991 m for men, 0.838 m for women; Senior: 1.067 m for men, 0.838 m for women). In addition, some athletes compete in the 400 mH event (U18: 0.838 m for men and 0.762 m for women; Senior: 0.914 m for men and 0.762 m for women) as well as short-sprint events. Thus, it is crucial to clarify the effect of hurdle height on the spatiotemporal variables affecting hurdle clearance.

Therefore, this study aimed to determine the effects of different hurdle heights on hurdle clearance style and important spatiotemporal variables that affect hurdle clearance performance, taking into account body height and interval limitations. This study hypothesized that different hurdle heights alter the spatiotemporal variables, which contribute significantly to the velocity during hurdle clearance.

## Methods

### Participants

The participants were 12 male college hurdlers (height, 1.75 ± 0.04 m; mass, 67.50 ± 4.35 kg) who had experience in either or both the 110 mH (personal record: 17.31 ± 1.73 s, *n* = 8) and 400 mH events (personal record: 53.79 ± 2.48 s, *n* = 8). The participants were informed of the study's purpose and the experimental procedure in advance and agreed to participate in the experiment. The experiment was conducted without discomfort to the participants in accordance with the Declaration of Helsinki. The local ethics review board approved the experimental protocol.

### Procedures

The experiments were conducted on a straight track of an all-weather track. The participants had to clear the hurdles set up at 15 m from the start at the fastest speed possible. The hurdle height was set at 10% higher (High: 1.063 ± 0.03 m) and 10% lower (Low: 0.870 ± 0.02 m) than the CM height (0.960 ± 0.02 m, 55.37 ± 0.28% of the height) of each participant. The absolute mean height of the hurdle was close to the hurdle height for the men's 110 mH event (1.067 m) in high condition. In contrast, absolute mean hurdle height in the Low condition was close to the hurdle height in the U18 men's 400 mH event (0.838 m). The trials were conducted using a standing start and while wearing spiked shoes.

Before the trials, participants had the option to warm up for a maximum of 30 min and practiced hurdle clearance in the experimental setting at least three times. Participants were instructed to take off on their ninth step from the starting point. When participants were forced to make large adjustments to their stride due to limitations in approach distance, the starting position was moved (range: 10–50 cm) to ensure a natural transition to takeoff. If any part of the body contacted the hurdle and the hurdle collapsed, or if the hurdler's takeoff or landing movement was disrupted such that there was a large deceleration, the trial was considered invalid. To avoid significant changes in sprint form after landing, the hurdlers were instructed to maintain their sprint form until the 30-m point after landing. The trials were performed at least twice at each height. All participants completed the trials within four attempts at each height, including the failed trials. A rest period of at least 5 min was allowed between trials. Hurdle clearance for successful trials was measured twice for both the High and Low conditions.

These experimental procedures were selected based on the assumption that hurdle clearance involving rapid acceleration with full effort was unfamiliar to the 400-m hurdlers participating in our study (Ozaki and Ueda, 2021). In addition, approach distances that are too long may affect the hurdler's ability to make stride adjustments, which can, in turn, affect takeoff technique due to increased stride accumulation error (Ozaki et al., 2019). Furthermore, previous studies have indicated that differences in runner height affect the spatiotemporal variables of hurdle clearance. Although partial correlation analysis has been used to assess changes in these variables for standard hurdle heights while controlling for height, it remains unclear whether the results can be applied to hurdlers of different heights (Nagahara et al., 2021). For example, given hurdles of the same height, a short hurdler may apply the High hurdle technique, while a tall hurdler may apply the low hurdle technique. Therefore, we considered these settings reasonable for evaluating individual hurdle clearance techniques, excluding the effects of interval, height, and stride adjustment ability.

### Experimental Setup

To calculate the CM height of the participants, they were positioned upright in the center of square markers placed 2.5 m apart on the ground, and their entire body was photographed with one camera from the front. To calibrate the aspect ratio of the images, a 2.0 m calibration pole was included both vertically and horizontally.

To construct a three-dimensional coordinate system from takeoff to landing, shooting ranges of 6.0 m in the direction of running, 1.2 m in the lane width, and 2.0 m in the lane height were set around the hurdle installation position. From both sides of the hurdle, six fixed high-speed cameras (EXILIM EX-ZR1700, CASIO, Tokyo, Japan, 240 frames/s) were used to film the entire body of the participants in each trial from takeoff to landing, diagonally in front and behind the right side and diagonally in front and behind the left side. Before the trial, a total of 30 control points were photographed with each camera: three in the direction of the participants' movement, two to the side, and five in the vertical direction.

### Video Analysis

The video images were captured on a personal computer, and a skilled examiner manually digitized 21 body feature points (head, tragion, upper margin of the sternum, shoulders, elbows, wrists, fingertips, greater trochanters, knees, ankles, heels, and toes) at 120 Hz using a video motion analysis system (Frame-DIAS V, DKH Retail Limited, Cheltenham, United Kingdom). Colored markers were attached to each participant's body points in advance so that their body points could be identified. The CM height of the participants was calculated using the two-dimensional four-point real-length conversion method based on the calibration performed. The coordinates of the participants' CM were obtained using the body part inertia coefficient of Ae (1996). All trials were digitized, including 10 frames before and after the touchdown of the takeoff foot to the release of the landing foot. Based on the digitized data, the actual coordinate values of each trial were obtained using the three-dimensional direct linear transformation method. The obtained three-dimensional coordinates were smoothed using a Butterworth digital filter after determining the optimal cutoff frequency using residual analysis (Wells, 1980). The cutoff frequencies were 6.00–13.32, 5.64–12.96, and 5.76–13.68 Hz in the front-back, left-right, and vertical directions, respectively. The coordinate data were projected in the sagittal plane and analyzed in the two-dimensional plane.

The reliability of digitizing each test was confirmed by intraclass correlation coefficients, where the same examiner digitized the same test twice. The interval between the digitization was more than 48 h (Paradisis and Cooke, 2001; Paradisis et al., 2019). The intraclass correlation coefficients of the variable values (described below) calculated by the digitization ranged from 0.888 to 0.999.

### Variables

Each calculated variable was the mean value of two trials. The variables calculated for this study were as follows:

#### Velocity

- Hurdle clearance velocity (HC-v): The mean horizontal velocity from touchdown on takeoff to release on landing during hurdle clearance.
- Sprint velocity: The mean horizontal velocity from touchdown at step-9 to release at step-10 in the sprint trial.
- HC-index: The value of HC-v divided by sprint velocity. This was used as a technical index for hurdle clearance, independent of sprint ability, and height.
- Rate of deceleration on takeoff (D-takeoff): The rate of decrease in horizontal velocity from right before touchdown on takeoff to immediately after release on takeoff.
- Rate of deceleration on landing (D-landing): The rate of decrease in horizontal velocity from right before touchdown on landing to immediately after release on landing.

In addition, the following variables were calculated as indicators to identify the characteristics of hurdlers with fast High against Low or fast Low against High:

- HC-high/low: The value obtained by dividing the HC-v of High by the HC-v of Low.
- HC-low/high: The value obtained by dividing the HC-v of Low by the HC-v of High.

#### Spatiotemporal Variables

**Figure 1:**
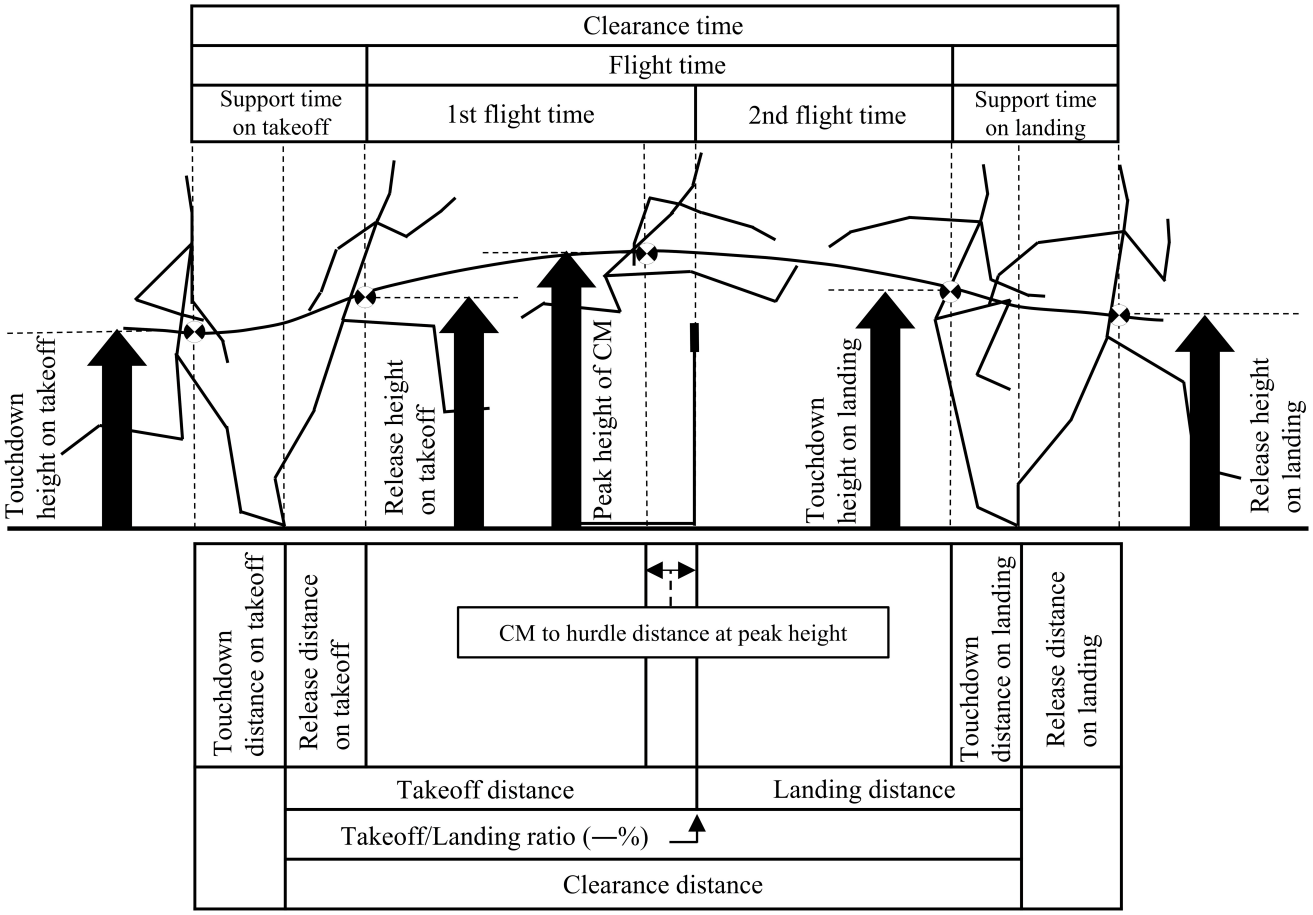
Spatiotemporal variables of hurdle clearance examined in this study. Adapted from Ozaki and Ueda (2021). Takeoff/Landing ratio: The takeoff distance divided by clearance distance. CM to hurdle distance at peak height: The horizontal distance between the hurdle and the CM at the peak height of the CM. The direction toward the front of the hurdle was defined as positive, and that toward the back of the hurdle was defined as negative. The above-mentioned spatial variables (CM height) were divided by the CM height of each participant and multiplied by 100 for analysis. The above-mentioned spatial variables (horizontal distance) were divided by the height of each participant and multiplied by 100 for analysis.

Figure 1 shows the spatiotemporal variables of hurdle clearance examined in this study.

### Statistical Analyses

Corresponding *t*-tests were conducted to examine the differences between each variable at High and Low levels. Effect sizes were defined as small (0.20–0.49), medium (0.50–0.79), large (0.80–1.19), very large (1.2–1.99), or huge (>2.0) according to Cohen's effect size (Cohen, 1988). Pearson's product rate correlation coefficients were calculated to examine the relationship between each variable and HC-v for High and Low, and HC-High/Low and HC-Low/High. In addition, to examine differences in the strength of the relationship between High and Low HC-v and each variable, tests for differences in the corresponding correlation coefficients were conducted for the correlation coefficients between High and Low HC-v and each variable. The *t*-test and correlation analysis were performed using SPSS statistics (version 20, IBM, Japan). The differences between the correlation coefficients were analyzed using the R statistical software package (R Foundation for Statistical Computing, Vienna, Austria). Variables were expressed as mean ± standard deviation. The significance level was set at <5%.

## Results

Table 1 shows the values of the variables at each hurdle height in the hurdle clearance and differences between the hurdle heights. In all hurdlers, there was deceleration on takeoff in both High and Low. However, on landing, horizontal velocity both decreased (High, *n* = 9; Low, *n* = 8) and increased (High, *n* = 3; Low, *n* = 4). Table 2 shows the relationship between HC-v and each variable and the difference in correlation coefficients between High and Low. Table 3 shows the relationship between HC-High/Low and HC-Low/High and each variable.

**Table 1:** Values of the variables during hurdle clearance (HC) at each hurdle height and differences between the two conditions.

**Variables**	**High (mean ±SD)**	**Low (mean ±SD)**	**Differences (*p*-value)**	**Cohen's *d***
HC-v (m ·s^−1^)	6.68 ± 0.36	7.17 ± 0.36	***p*** **<** **0.001**	1.315
D-takeoff (%)	12.23 ± 2.52	7.58 ± 2.31	***p*** **<** **0.001**	1.857
D-landing (%)	1.86 ± 2.63	0.84 ± 1.61	*p* = 0.208	0.451
Support time on takeoff (s)	0.14 ± 0.01	0.13 ± 0.01	***p*** **=** **0.011**	0.427
1st flight time (s)	0.23 ± 0.02	0.20 ± 0.03	***p*** **<** **0.001**	0.941
2nd flight time (s)	0.27 ± 0.02	0.20 ± 0.04	***p*** **<** **0.001**	2.131
Support time on landing (s)	0.11 ± 0.02	0.10 ± 0.01	***p*** **=** **0.001**	0.811
Flight time (s)	0.49 ± 0.03	0.40 ± 0.04	***p*** **<** **0.001**	2.482
Clearance time (s)	0.74 ± 0.05	0.63 ± 0.06	***p*** **<** **0.001**	2.024
Takeoff distance (%)	106.41 ± 9.7	107.72 ± 9.81	*p* = 0.484	0.130
Landing distance (%)	113.4 ± 7.97	92.90 ± 11.86	***p*** **<** **0.001**	1.960
Clearance distance (%)	219.81 ± 11.71	200.62 ± 10.91	***p*** **<** **0.001**	1.637
Takeoff/landing ratio (%)	48.37 ± 3.06	53.76 ± 4.93	***p*** **=** **0.002**	1.269
Touchdown height on takeoff (%)	94.79 ± 2.40	95.70 ± 2.29	***p*** **=** **0.015**	0.392
Release height on takeoff (%)	117.32 ± 1.69	113.82 ± 1.71	***p*** **<** **0.001**	2.075
Touchdown height on landing (%)	114.77 ± 3.07	113.28 ± 2.87	***p*** **<** **0.001**	0.505
Release height on landing (%)	99.56 ± 4.06	102.53 ± 3.80	***p*** **<** **0.001**	0.763
Peak height of the CM (%)	145.78 ± 3.11	133.19 ± 4.30	***p*** **<** **0.001**	3.387
Touchdown distance on takeoff (%)	32.77 ± 3.26	29.44 ± 3.87	***p*** **<** **0.001**	0.898
Release distance on takeoff (%)	20.36 ± 2.05	24.32 ± 2.94	***p*** **<** **0.001**	1.510
Touchdown distance on landing (%)	12.04 ± 3.63	11.73 ± 3.16	*p* = 0.825	0.089
Release distance on landing (%)	29.23 ± 3.82	28.02 ± 3.45	*p* = 0.194	0.320
CM-to-hurdle distance at peak height (%)	−7.76 ± 7.30	0.70 ± 10.54	***p*** **=** **0.011**	0.901

*HC-v, hurdle clearance velocity; D-takeoff; rate of deceleration on takeoff; D-landing, rate of deceleration on landing; CM, center of mass*.

**Table 2:** Relationship between hurdle clearance velocity (HC-v) and each variable in the high and low conditions and differences in correlation coefficients.

**Variables**	**HC-v (high)**	**HC-v (low)**	**Difference in correlation coefficient (*p*-value)**
D-takeoff	−0.761**	−0.553	*p* = 0.261
D-landing	−0.104	−0.652*	*p* = 0.105
Support time on takeoff	−0.629*	−0.476	*p* = 0.417
1st flight time	−0.009	−0.530	***p*** **=** **0.037**
2nd flight time	−0.684*	−0.370	*p* = 0.207
Support time on landing	−0.486	−0.475	*p* = 0.964
Flight time	−0.608*	−0.751**	*p* = 0.363
Clearance time	−0.682*	−0.789**	*p* = 0.443
Takeoff distance	0.484	0.172	*p* = 0.218
Landing distance	0.424	−0.164	*p* = 0.227
Clearance distance	0.690*	−0.023	***p*** **=** **0.029**
Takeoff/landing ratio	0.155	0.195	*p* = 0.921
Touchdown height on takeoff	0.335	0.186	*p* = 0.536
Release height on takeoff	−0.771**	−0.723**	*p* = 0.784
Touchdown height on landing	0.017	0.268	*p* = 0.304
Release height on landing	0.365	0.577*	*p* = 0.321
Peak height of the CM	−0.743**	−0.743**	*p* = 1.000
Touchdown distance on takeoff	−0.522	−0.541	*p* = 0.921
Release distance on takeoff	0.626*	0.701*	*p* = 0.701
Touchdown distance on landing	0.079	0.305	*p* = 0.606
Release distance on landing	−0.078	−0.200	*p* = 0.698
CM-to-hurdle distance at peak height	−0.270	−0.171	*p* = 0.785

*p < 0.05, **p <0.01.

*HC-v, hurdle clearance velocity; D-takeoff; rate of deceleration on takeoff; D-landing, rate of deceleration on landing; CM, center of mass*.

**Table 3:** Relationship of HC-high/low and HC-low/high with each variable.

**Variables**	**HC-high/low (vs. high variables)**	**HC-low/high (vs. low variables)**
D-takeoff	−0.379	−0.019
D-landing	0.105	−0.157
Support time on takeoff	−0.265	0.187
1st flight time	0.572	−0.477
2nd flight time	−0.217	0.339
Support time on landing	−0.350	0.506
Flight time	0.084	−0.136
Clearance time	−0.140	0.048
Takeoff distance	0.627*	−0.508
Landing distance	−0.060	0.421
Clearance distance	0.479	−0.0001
Takeoff/landing ratio	0.514	−0.504
Touchdown height on takeoff	−0.031	−0.043
Release height on takeoff	−0.569	−0.007
Touchdown height on landing	−0.531	0.628*
Release height on landing	−0.275	0.104
Peak height of the CM	−0.282	0.048
Touchdown distance on takeoff	−0.137	0.117
Release distance on takeoff	0.262	0.215
Touchdown distance on landing	−0.267	0.306
Release distance on landing	−0.038	0.315
CM to hurdle distance at peak height	0.217	−0.537

* *p < 0.05*.

*HC, hurdle clearance; D-takeoff; rate of deceleration on takeoff; D-landing, rate of deceleration on landing; CM, center of mass*.

## Discussion

In this study, we examined the effects of different hurdle heights on the spatiotemporal variables of hurdle clearance, taking into account the limitations of height and interval. Our results indicated that changes in hurdle height led to changes in spatiotemporal variables during hurdle clearance. Notably, differences in hurdle height also altered the spatiotemporal variables that are determinants of HC-v. These findings support our hypothesis and may be useful for coaches who train hurdlers of different heights and those for whom hurdle height varies due to age group or event.

### Differences in Spatiotemporal Variables in Hurdle Clearance by Different Hurdle Heights

As a result of examining the differences in spatiotemporal variables between High and Low, significant differences were found among the variables, except takeoff distance, touchdown distance on landing, release distance on landing, and D-landing. In particular, the effect sizes for 2nd flight time, flight time, clearance time, release height on takeoff, peak height of the CM, and landing distance were huge. Therefore, in High, which required a higher CM rise, the hurdler completed the takeoff at a higher CM height than in Low (i.e., large takeoff angle) and landed farther from the hurdle because of the higher CM and longer flight time required.

Although there was a difference in the release height on takeoff, there was no significant difference in takeoff distance by hurdle height. Theoretically, it is expected that a low hurdle height will allow for a longer takeoff distance because the hurdle height can be maintained at a higher horizontal velocity. Alternatively, in High, the release height on the takeoff and the top of the CM are both higher; thus the flight time is longer. Therefore, even though the loss of horizontal velocity will be large, the hurdler may still be able to obtain a long takeoff distance at High, as long as the CM vertex does not shift excessively to the back of the hurdle. Thus, some factors may lengthen the takeoff distance for each of the low and high hurdles. As such, factors other than hurdle height (e.g., intervals, sprints, and body size differences between men and women) may have contributed to the discrepancies in the results of previous studies (Salo et al., 1997; González-Frutos et al., 2019; Hanley et al., 2021) that examined differences in takeoff distance between men and women. The lack of a difference in takeoff distance between the High and Low conditions in our study may have been due to the experimental setting, which was not limited by the interval.

Alternatively, touchdown height on landing was significantly higher, while release height on landing was significantly lower in High landings. In the High group, the hurdler landed at a higher CM height to quickly mitigate the larger downward velocity in landings with more extended lead leg, thus requiring a larger CM downward distance. In fact, male hurdlers have a greater lead leg knee angle at landing from higher hurdles than female hurdlers (Hanley et al., 2021). Despite this, there was no significant difference in D-landing between the High and Low groups. In this regard, McLean (1994) emphasized that the touchdown distance on landing should be close to the CM to reduce the deceleration on landing. As touchdown distance on landing and release distance on landing were not significantly different; therefore, the difference in hurdle height was unlikely to affect touchdown distance and release distance on landing, and the effect of CM descent velocity on D-landing was considered small. However, because the downward movement of the CM continues until one step after landing (recovery step; McDonald and Dapena, 1991a), negative effects, including the recovery step after landing, may occur. Furthermore, it has been reported that an increase in running speed during this recovery step may be associated with improved sprint hurdle records (Nagahara et al., 2021). Because this study did not include the recovery step in its analysis, further research with a wider range of analyses is required.

The CM to hurdle distance at peak height shifted to the back of the hurdle, and the landing distance became longer at High. Salo et al. (1997) reported that the CM to hurdle distance at peak height is further in front of the hurdle in women than in men and stated that the cause of this is not completely known. In addition, the peak height of the CM should be in front of the hurdle, particularly for female hurdlers, to enable a quick transition to landing motion (Bedini, 2016). The participants in this study included 400 mH but were not highly experienced. Therefore, High, poorly skilled hurdlers were likely to shift the CM to hurdle distance at peak height to the back of the hurdle by excessively increasing vertical velocity to ensure that they crossed the hurdle higher than the CM. In addition, when the CM to hurdle distance at the peak height is reached in front of the hurdle, it inevitably leads to the CM crossing the hurdle with a downward movement. To avoid contact with the hurdle, sufficient vertical space between the hurdle and the CM is required. However, in the High group, a large CM rise is required, and if the athlete tries to raise the CM excessively to avoid hitting the hurdle, it may lead to an even larger deceleration on takeoff. Therefore, when the hurdle is higher than the CM, or in the case of a short hurdler, the coach may wish to instruct the athlete not to shift the peak height of the CM to the front of the hurdle.

### Important Spatiotemporal Variables Related to Hurdle Clearance Velocity in Hurdles of Different Heights

Similar variables that showed significant correlations between High and Low were flight time, clearance time, release height on takeoff, peak height of CM, and release distance on takeoff. Keeping the CM height low and placing the CM further forward in takeoff is a reasonable mechanism to maintain horizontal velocity while suppressing CM rise. Therefore, the low angle of the CM during takeoff, low CM peak, and associated short flight time are important variables for increasing clearance velocity, regardless of hurdle height.

The variables that showed a significant correlation with High were D-takeoff, support time on takeoff, 2nd flight time, and clearance distance. There was a significant difference in the correlation coefficients for clearance distance. In addition, takeoff distance was significantly correlated with HC-High/Low.

High requires a higher CM increase. To reduce the takeoff angle and deceleration, a high running velocity and a long takeoff distance are required. In contrast, attempting to achieve a small takeoff angle at a low running velocity does not result in a sufficient CM height. In addition, during hurdle clearance, if the running velocity is low, the amount of elastic energy stored in the legs is insufficient, the ground contact time must, therefore, be extended, and additional positive work must be performed by the muscles to obtain a higher CM height (Mauroy et al., 2013). Furthermore, runners with higher running velocities have shorter ground contact times during sprinting and higher vertical stiffness during ground contact (Rabita et al., 2015; Nagahara and Zushi, 2017; Paradisis et al., 2019). In addition, a higher running velocity causes more angular momentum available for swinging the lead leg in hurdle clearance, which allows for quicker landing (McDonald and Dapena, 1991b). Therefore, a higher running velocity was also considered a factor that could explain the shorter 2nd flight time in High. These results suggest that, when training athletes to clear high hurdles or shorter athletes, coaches may need to focus on improving sprinting ability and elastic muscle strength to obtain longer clearance distances.

D-landing and release height on landing showed significant correlations in Low. A significant difference in the correlation coefficient was found for 1st flight time, but no significant correlation was found with HC-v (Low). Touchdown height during landing was significantly correlated with HC-low/high. Thus, the variables affected by HC-v at Low were relatively more related to landing movements.

The difference in the takeoff technique did not affect HC-v because the takeoff technique was relatively easy in Low as it did not require a large increase in CM compared to that in High. On the other hand, because the peak of CM is low in Low, a quicker transition to landing is inevitable. Therefore, the difference in HC-v (Low) was more likely to be reflected by a quick landing technique with a smaller deceleration than in High. However, the correlation between HC-v (Low) and 2nd flight time and landing distance in Low was very weak, although quick landing was required in Low. In this regard, women attempting to clear low hurdles may be forced into landing with a low CM if the clearance CM is excessively low (McDonald and Dapena, 1991a; Hanley et al., 2021). Nagahara et al. (2021) also highlighted the importance of minimizing support time by raising the CM during toe-stand landings. Therefore, coaches may need to prioritize high CM landings in tall hurdlers and those required to clear low hurdles, rather than overly restricting CM increases during hurdle clearance or shortening flight time and landing distance.

### Limitation

This study covered from takeoff to landing in relation to hurdle clearance. The elevation and downward movement of the CM continues from the preparatory step one step before takeoff to the recovery step one step after landing (McDonald and Dapena, 1991a). Therefore, different hurdle heights during the preparation and recovery steps may lead to differences in technique (Hanley et al., 2021). In particular, since takeoff is easily influenced by the preparation step, extensive analysis that includes preparation and recovery steps is necessary to clarify the detailed technique differences between different hurdle heights. In addition, this study did not consider the interval limitations. However, we analyzed a single hurdle clearance in a 15-m approach run, hurdlers might not be able to accelerate sufficiently and might have behaved differently with respect to actual hurdle clearance during a race. The scope of analysis and control for other factors, such as intervals, should be expanded to enhance coaching during practice.

## Conclusion

In this study, we examined the effects of different hurdle heights on the spatiotemporal variables of hurdle clearance, taking into account the effects of interval limitations and differences in height. Our results indicated that different hurdle heights altered not only spatiotemporal variables during hurdle clearance, but also spatiotemporal variables related to HC-v. In particular, low D-takeoff and long clearance distance were associated with HC-v in the High condition. In the Low condition, high CM during landing and low D-landing was associated with HC-v. Therefore, coaches should consider not only the differences in spatiotemporal variables caused by different hurdle heights, but also the spatiotemporal variables associated with HC-v at each height. Further, the results of this study suggest that the spatiotemporal variables to focus on during training may differ depending on the height of the hurdler, even for the same hurdle height.

## Data Availability Statement

The raw data supporting the conclusions of this article will be made available by the authors, without undue reservation.

## Ethics Statement

The studies involving human participants were reviewed and approved by Joint Committee for Ethical Review of the Education Program, Graduate School of Human and Social Sciences. The patients/participants provided their written informed consent to participate in this study.

## Author Contributions

YO drafted the article and its critical revision, interpreted the results of the research, and approved the final version to be published. All authors conceptualized and designed the study and performed data collection.

## Conflict of Interest

The authors declare that the research was conducted in the absence of any commercial or financial relationships that could be construed as a potential conflict of interest.

## Publisher's Note

All claims expressed in this article are solely those of the authors and do not necessarily represent those of their affiliated organizations, or those of the publisher, the editors and the reviewers. Any product that may be evaluated in this article, or claim that may be made by its manufacturer, is not guaranteed or endorsed by the publisher.
